# Supplementary material for: Co-Surfactant-Free Bioactive Protein Nanosheets for the Stabilization of Bioemulsions Enabling Adherent Cell Expansion
Source: Biomacromolecules. 2023 Jan 23;24(10):4465–77. doi: 10.1021/acs.biomac.2c01289 (PMC10565825; doi:10.1021/acs.biomac.2c01289)
Supplement: Supplementary file 1 — bm2c01289_si_001.pdf [file bm2c01289_si_001.pdf]

Co-Surfactant-Free Bioactive Protein Nanosheets for the Stabilisation of Bioemulsions Enabling Adherent Cell Expansion

Supplementary Information

Alexandra Chrysanthou[1,2], Minerva Bosch-Fortea[1,2] and Julien E. Gautrot[1,2*]

[1] Institute of Bioengineering and [2]School of Engineering and Materials Science, Queen Mary, University of London, Mile End Road, London, E1 4NS, UK.

[*]Corresponding author. j.gautrot@qmul.ac.uk

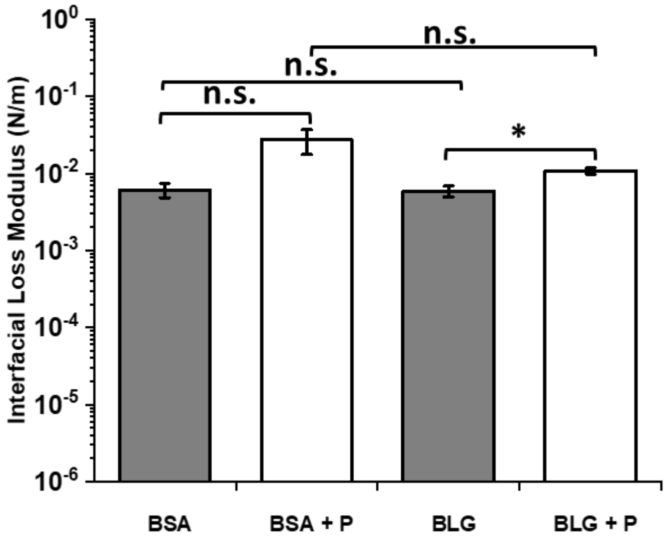

Supplementary Figure S1. Interfacial shear loss modulus of BSA, BSA + PFBC, BLG, BLG+PFBC, casein and casein + PFBC at oscillating amplitude 10^{-4} rad and frequency of 1 Hz. Error bars are s.e.m; n=3.

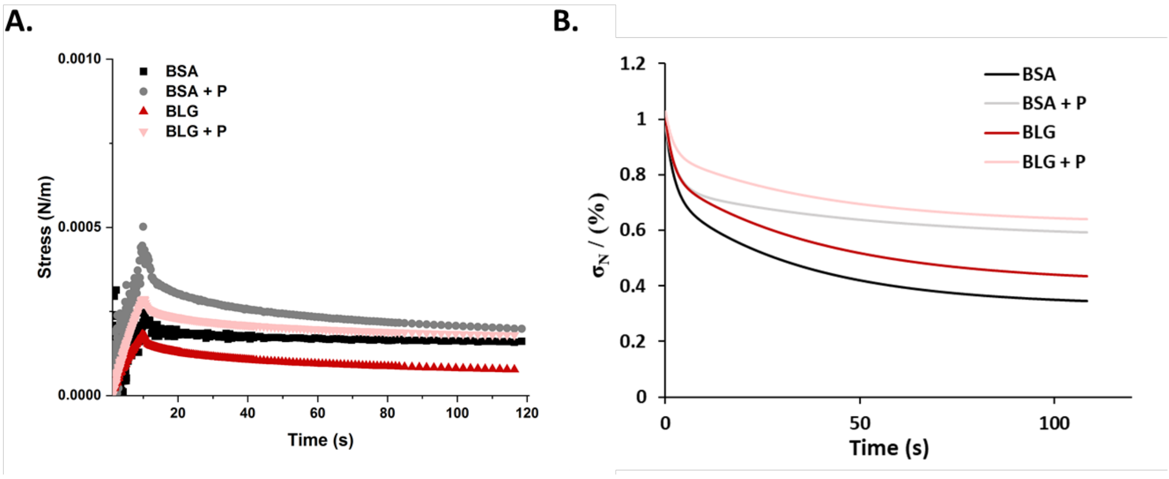

Supplementary Figure S2. Stress relaxation experiments carried out on protein nanosheets formed at liquid-liquid interfaces with and without PFBC (10 μg/mL). Data is shown as normalised stress (σ_N) extracted from stress relaxation experiments at a strain 0.5%.

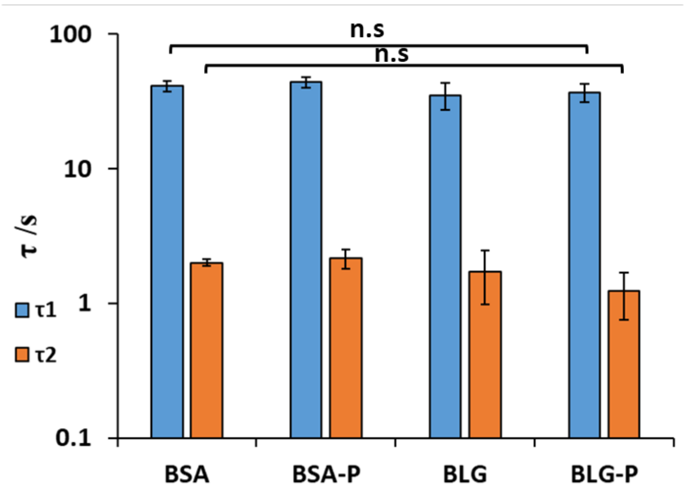

Supplementary Figure S3. Characterisation of the stress relaxation profiles associated with protein nanosheets studied. Data were extracted from stress relaxation at a strain of 0.5%. (A) Comparing BSA, BLG and casein (all at 1 mg/mL) with and without PFBC. Error bars are s.e.m; n=3.

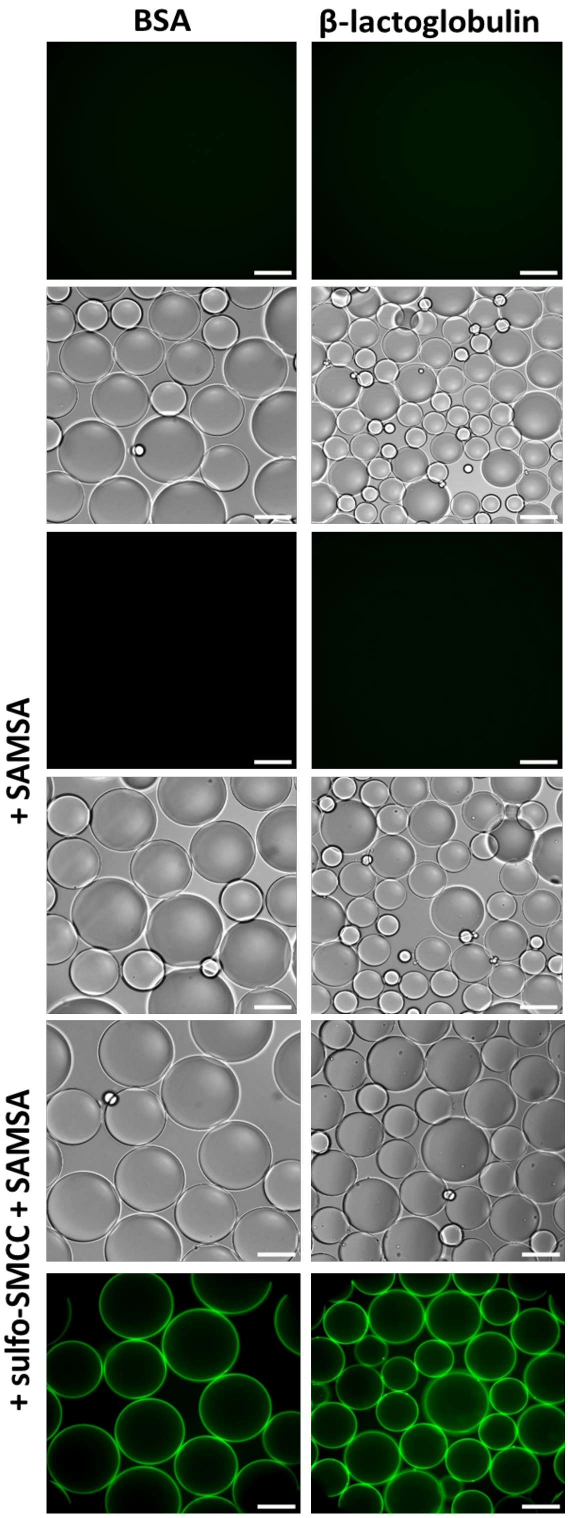

Supplementary Figure S4. Epifluorescence microscopy images were SAMSA fluorescein (green, AB F3648) conjugated. Scale bars, 200 μm.

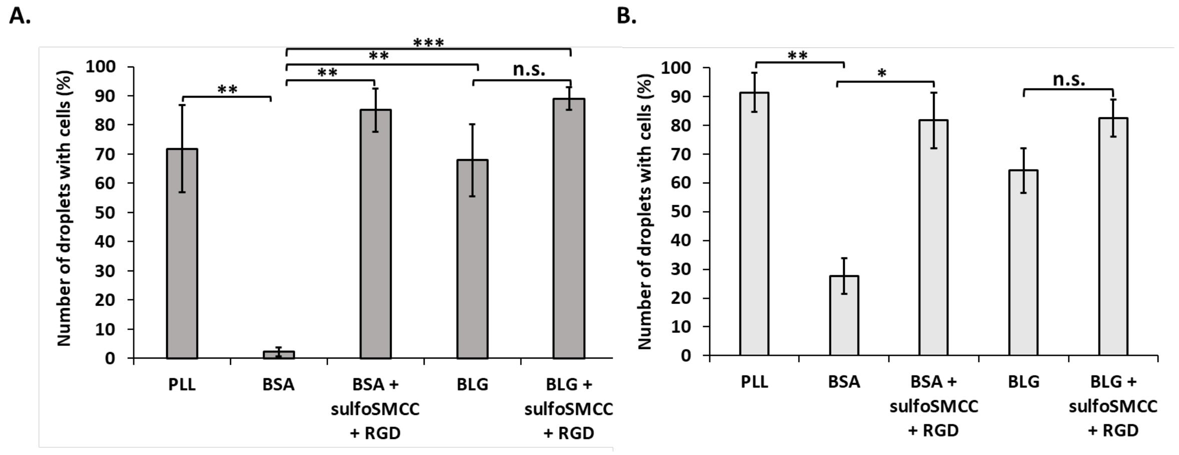

Supplementary Figure S4. Percentage number of droplets that were covered with (A) MSCs and (B) HEK293T.

Supplementary Tables

Supplementary Table S1 Summary of statistical analysis of storage modulus data obtained by frequency sweep after the proteins were adsorbed at fluorinated-PBS interfaces with and without co-surfactant (PFBC) at 10 μg/ mL .

	MeanDiff	Prob	
**BSA + PFBC BSA**	0.11869	0.14564	**n.s**
**BLG + PFBC BLG**	0.04136	0.00209	**
**Casein + PFBC Casein**	0.10352	0.03876	*
**BLG BSA**	-0.00383	0.88433	**n.s**
**Casein BSA**	-0.0356	0.0104	*
**Casein BLG**	-0.03177	0.01747	*
**BLG + PFBC BSA + PFBC**	-0.08115	0.42242	**n.s**
**Casein + PFBC BSA + PFBC**	-0.05077	0.69215	**n.s**
**Casein + PFBC BLG + PFBC**	0.03038	0.8718	**n.s**

Supplementary Table S2 Summary of statistical analysis of storage modulus data obtained by frequency sweep after the proteins were adsorbed at fluorinated-PBS interfaces with and without co-surfactant (PFBC) at 10 μg/ mL.

	MeanDiff	Prob	
**BSA + PFBC BSA**	0.0211	0.09442	**n.s**
**BLG + PFBC BLG**	0.00488	0.02317	*
**Casein + PFBC Casein**	0.03089	0.01316	*
**BLG BSA**	-2.20E-04	0.9853	**n.s**
**Casein BSA**	-0.00564	0.01322	*
**Casein BLG**	-0.00542	0.01583	*
**BLG + PFBC BSA + PFBC**	-0.01644	0.29054	**n.s**
**Casein + PFBC BSA + PFBC**	0.00415	0.90799	**n.s**
**Casein + PFBC BLG + PFBC**	0.02059	0.1718	**n.s**

Supplementary Table S3 Summary of statistical analysis of data obtained from stress relaxation experiments at a strain of 0.5 % .

	MeanDiff	Prob	
**BSA-P BSA**	22.55267	0.09127	**n.s**
**BLG-P BLG**	11.965	0.06436	**n.s**
**BLG-P BSA-P**	-2.98533	0.9436	**n.s**
**Casein-P BSA-P**	-26.076	0.06569	**n.s**

**Casein-P BLG-P**	-23.0907	0.00896	**
**BLG BSA**	7.60233	0.01709	*
**BLG BSA-P**	-14.9503	0.21667	**n.s**

Supplementary Table S4 Summary of statistical analysis of data of τ_1 values obtained from stress relaxation experiments at a strain of 0.5 %.

	**MeanDiff**	**Prob**	
**t1 BLG t1 BSA**	1.48429	0.99375	n.s
**t1 BSA-P t1 BSA**	5.88717	0.74876	n.s
**t1 BSA-P t1 BLG**	4.40288	0.87215	n.s
**t1 BLG-P t1 BSA**	8.50963	0.50131	n.s
**t1 BLG-P t1 BLG**	7.02534	0.64101	n.s
**t1 BLG-P t1 BSA-P**	2.62246	0.96783	n.s

Supplementary Table S5 Summary of statistical analysis of data of τ_2 values obtained from stress relaxation experiments at a strain of 0.5 %.

	**MeanDiff**	**Prob**	
**t2 BLG t2 BSA**	-0.49612	0.84427	n.s
**t2 BSA-P t2 BSA**	0.76719	0.60681	n.s
**t2 BSA-P t2 BLG**	1.26331	0.23655	n.s
**t2 BLG-P t2 BSA**	0.92303	0.46832	n.s
**t2 BLG-P t2 BLG**	1.41916	0.16735	n.s
**t2 BLG-P t2 BSA-P**	0.15585	0.99356	n.s

Supplementary Table S6 Summary of statistical analysis of data obtained from the SPR data for the protein binding (1 mg/mL) at the perfluorodecanethiol pre-treated chips.

	**MeanDiff**	**Prob**	
**β-lactoglobulin BSA**	461.2667	0.45494	**n.s**
**β-casein BSA**	1184.367	0.03828	*
**β-casein β-lactoglobulin**	723.1	0.19123	**n.s**

Supplementary Table S7 Summary of statistical analysis of data obtained from the SPR data for the sulfo-SMCC (2 mg/mL) at the surface of protein layers (BSA, BLG and casein).

	**MeanDiff**	**Prob**	
**β-lactoglobulin BSA**	105.0667	0.93711	**n.s**
**β-casein BSA**	-80.7333	0.9622	**n.s**
**β-casein β-lactoglobulin**	-185.8	0.81995	**n.s**

Supplementary Table S8 Summary of statistical analysis of data obtained from the SPR data for the RGD (1.6 mg/mL) at the surface of the sulfo-SMCC layer.

	MeanDiff	Prob	
**β-lactoglobulin BSA**	26.06667	0.88869	**n.s**
**β-casein BSA**	47.4	0.68847	**n.s**
**β-casein β-lactoglobulin**	21.33333	0.92352	**n.s**

Supplementary Table S9 Summary of statistical analysis of data obtained from the epifluorescence images for the SAMSA-flurorescein binding on BSA, BLG and casein emulsion droplets in the presence and absence of sulfo-SMCC.

	MeanDiff	Prob	
**BLG BSA**	-23.5313	0.93412	**n.s**
**Casein BSA**	-37.1652	0.8461	**n.s**
**Casein BLG**	-13.6339	0.97721	**n.s**
**BLG BSA**	-23.5313	0.99966	**n.s**
**Casein BSA**	-37.1652	0.997	**n.s**
**Casein BLG**	-13.6339	0.99998	**n.s**
**BSA + SAMSA BSA**	127.9757	0.63783	**n.s**
**BSA + SAMSA BLG**	151.507	0.47613	**n.s**
**BSA + SAMSA Casein**	165.1409	0.39065	**n.s**
**BLG + SAMSA BSA**	6.54822	1	**n.s**
**BLG + SAMSA BLG**	30.07956	0.9989	**n.s**
**BLG + SAMSA Casein**	43.71344	0.99363	**n.s**
**BLG + SAMSA BSA + SAMSA**	-121.427	0.68331	**n.s**
**Casein+ SAMSA BSA**	40.45822	0.99554	**n.s**
**Casein+ SAMSA BLG**	63.98956	0.96608	**n.s**
**Casein+ SAMSA Casein**	77.62344	0.9267	**n.s**
**Casein+ SAMSA BSA + SAMSA**	-87.5174	0.88595	**n.s**
**Casein+ SAMSA BLG + SAMSA**	33.91	0.99805	**n.s**

**BLG + sulfo-SMCC + SAMSA BSA + sulfo-SMCC + SAMSA**	-649.481	0.53284	**n.s**
**Casein+ sulfo-SMCC + SAMSA BSA + sulfo-SMCC + SAMSA**	96.22956	0.98474	**n.s**
**Casein+ sulfo-SMCC + SAMSA BLG + sulfo-SMCC + SAMSA**	745.7107	0.44729	**n.s**
**BSA + sulfo-SMCC + SAMSA BSA**	4146.55	8.79E-04	***
**BLG + sulfo-SMCC + SAMSA BLG**	3520.6	2.10E-04	***
**Casein+ sulfo-SMCC + SAMSA Casein**	4279.945	7.43E-04	***

Supplementary Table S10 Summary of statistical analysis of data obtained from cell (MSCs) on emulsion droplets after seven days in culture.

	**MeanDiff**	**Prob**	
**PLL TCP**	-17544.4	0.36446	n.s
**BSA TCP**	-75266.7	1.52E-05	***
**BSA PLL**	-57722.2	2.16E-04	***
**BLG TCP**	-30788.9	0.03204	***
**BLG PLL**	-13244.4	0.63719	n.s
**BLG BSA**	44477.78	0.00222	**
**BSA +sulfo-SMCC + RGD TCP**	-29829.6	0.03876	*
**BSA +sulfo-SMCC + RGD PLL**	-12285.2	0.70135	n.s
**BSA +sulfo-SMCC + RGD BSA**	45437.04	0.00186	**
**BSA +sulfo-SMCC + RGD BLG**	959.2593	1	n.s
**BLG +sulfo-SMCC + RGD TCP**	-15144.4	0.50989	n.s
**BLG +sulfo-SMCC + RGD PLL**	2400	0.99969	n.s
**BLG +sulfo-SMCC + RGD BSA**	60122.22	1.46E-04	***
**BLG +sulfo-SMCC + RGD BLG**	15644.44	0.47765	n.s
**BLG +sulfo-SMCC + RGD BSA +sulfo-SMCC + RGD**	14685.19	0.54015	n.s

Supplementary Table S11 Summary of statistical analysis of data obtained from cell (HEK293T) on emulsion droplets after seven days in culture.

	**MeanDiff**	**Prob**	
**BSA +sulfo-SMCC + RGD BSA**	284000	0.0242	*
**BLG +sulfo-SMCC + RGD BLG**	297944.4	0.02759	*
**BSA +sulfo-SMCC + RGD TCP**	-146056	0.25486	**n.s**
**BLG +sulfo-SMCC + RGD TCP**	-148444	0.24588	**n.s**
**BLG +sulfo-SMCC + RGD BSA +sulfo-SMCC + RGD**	-2388.89	0.99953	**n.s**

Supplementary Table S12 Summary of statistical analysis of data obtained from the number of droplets covered with cells (MSCs) on emulsion droplets after seven days in culture.

	MeanDiff	Prob	
**BSA PLL**	-69.5612	0.00242	**
**BSA + sulfoSMCC + RGD PLL**	6.0635	0.98904	**n.s**
**BSA + sulfoSMCC + RGD BSA**	75.62472	0.00129	**
**BLG PLL**	-3.88011	0.99801	**n.s**
**BLG BSA**	65.68111	0.00367	**
**BLG BSA + sulfoSMCC + RGD**	-9.94361	0.93631	**n.s**
**BLG + sulfoSMCC + RGD PLL**	17.19101	0.689	**n.s**
**BLG + sulfoSMCC + RGD BSA**	86.75223	4.32E-04	***
**BLG + sulfoSMCC + RGD BSA + sulfoSMCC + RGD**	11.12751	0.90814	**n.s**
**BLG + sulfoSMCC + RGD BLG**	21.07113	0.52247	**n.s**

Supplementary Table S13 Summary of statistical analysis of data obtained from the number of droplets covered with cells (MSCs) on emulsion droplets after seven days in culture.

	MeanDiff	Prob	
**BSA PLL**	-63.759	0.00328	**
**BSA + sulfoSMCC + RGD PLL**	-9.69143	0.932	**n.s**
**BSA + sulfoSMCC + RGD BSA**	54.06757	0.01017	*
**BLG PLL**	-27.1195	0.26481	**n.s**
**BLG BSA**	36.63949	0.08696	**n.s**
**BLG BSA + sulfoSMCC + RGD**	-17.4281	0.64397	**n.s**
**BLG + sulfoSMCC + RGD PLL**	-8.80211	0.95079	**n.s**
**BLG + sulfoSMCC + RGD BSA**	54.9569	0.00914	**
**BLG + sulfoSMCC + RGD BSA + sulfoSMCC + RGD**	0.88933	0.99999	**n.s**
**BLG + sulfoSMCC + RGD BLG**	18.31741	0.60368	**n.s**
